# Plasma, Urinary, and Salivary Total Redox Status in Critically Ill Patients with Brain Injury and Secondary Organ Failure

**DOI:** 10.3390/antiox15020185

**Published:** 2026-02-02

**Authors:** Ewa Rynkiewicz-Szczepanska, Urszula Kosciuczuk, Katarzyna Anikiej, Anna Zalewska, Małgorzata Żendzian-Piotrowska, Mateusz Maciejczyk

**Affiliations:** 1 Department of Anesthesiology and Intensive Therapy, Medical University of Bialystok, Kilinskiego Street 1, 15-276 Bialystok, Poland; ewaryn@op.pl (E.R.-S.);; 2 Students Scientific Club “Biochemistry of Civilization Diseases”, Department of Hygiene, Epidemiology and Ergonomics, Medical University of Bialystok, Kilinskiego Street 1, 15-276 Bialystok, Poland; 3 Independent Laboratory of Experimental Dentistry, Medical University of Bialystok, Kilinskiego Street 1, 15-276 Bialystok, Poland; 4 Department of Hygiene, Epidemiology, and Ergonomics, Medical University of Bialystok, Kilinskiego Street 1, 15-276 Bialystok, Poland

**Keywords:** biofluids, brain, oxidative stress

## Abstract

Little is known about the clinical utility of blood, salivary, and urinary redox biomarkers in critically ill patients with brain injury and secondary organ failure. The aim of the study was to explore total antioxidant and oxidant status in neurocritically ill patients using ferric reducing antioxidant power (FRAP), total antioxidant capacity (TAC), and total oxidant status (TOS) in plasma, saliva, and urine from the study (n = 45) and the healthy control group (n = 49). We analyzed the relationship between well-known biomarkers of organ function and redox status in different biofluids. Plasma FRAP was significantly higher (*p* < 0.05), but salivary and urinary FRAP were statistically lower in the study group (*p* < 0.05, *p* < 0.001) compared with controls. The salivary and urinary TAC were statistically lower (*p* < 0.05, *p* < 0.001), while plasma TOS was significantly higher (*p* < 0.05) in the study group compared with the control group. Circulating redox status did not differ between survivors and non-survivors. Significant associations were observed in non-survivors: salivary TAC correlated with urea and creatinine; salivary FRAP with creatinine, troponin, and CRP; urinary TAC with troponin and P_a_O_2_/FiO_2_ ratio, as well as plasma FRAP with P_a_O_2_/FiO_2_ ratio. The plasma FRAP had a significant effect on survival (AUC = 0.687, *p* = 0.02), with 69% sensitivity and 83% specificity. Crucial differences in redox status in blood, saliva, and urine were observed between neurocritically ill patients and healthy controls; however, none of the biomarkers differed between survivors and non-survivors. Oxidative and antioxidant status correlated with organ function in non-survivors.

## 1. Introduction

Oxidative stress is an imbalance in the homeostasis between oxidants and antioxidants, with a predominance of oxidative processes [1,2,3,4,5]. The literature indicates that oxidative stress plays a crucial role in the development of chronic diseases such as diabetes, renal failure, heart failure, allergies, neuropsychiatric diseases, and carcinogenesis [6,7,8,9,10]. In sepsis, pathogen invasion triggers systemic immunological and inflammatory responses, leading to the activation of T lymphocytes, B lymphocytes, NK cells, and monocytes, which enhance reactive oxygen species (ROS) production and cytokine secretion. ROS overproduction, combined with limited antioxidant capacity and impaired oxygen utilization, increases protein and lipid peroxidation, leading to mitochondrial dysfunction and membrane damage in neurons and glial cells [11,12,13,14,15,16].

The intensity of oxidative stress can be measured using various markers, including oxidized proteins (advanced glycation end products, AGEs), protein carbonyls, advanced oxidation protein products (AOPPs), lipids (malondialdehyde, MDA), isoprostanes (F2-IsoPs), alkenals, alkadienals, 4-hydroxy-2-nonenal (HNE), and nucleic acids (deoxyguanosine) [17,18,19,20,21,22,23,24,25]. Given the complexity of redox reactions, it is challenging to determine oxidative stress solely from assessments of individual oxidation products or antioxidants. Therefore, measurements of total antioxidant capacity (TAC) and total oxidative status (TOS) are often used. It is well known that assessing the ROS-scavenging capacity or the total oxidant load in a sample provides more information about redox homeostasis than individual biomarkers. Plasma, serum, and blood cells (leukocytes and erythrocytes) are the most popular bioliquids for redox assays. The use of urine and saliva is a recent diagnostic trend due to their easy and noninvasive collection [22,23,24,25,26,27,28]. However, little is known about redox homeostasis in urine and saliva, particularly whether these bioliquids can be used to assess systemic antioxidant and oxidative status [29,30,31,32].

Knowledge about the role of oxidative stress in critically ill patients with secondary brain injury is also limited. The most well-described is redox status in sepsis. Serum TAC levels in patients with severe sepsis are positively correlated with organ failure classification based on Acute Physiology and Chronic Health Evaluation II (APACHE II) scores. Moreover, after controlling for age and serum creatinine level, TAC remained positively associated with APACHE II scores [20,21,22]. However, little is known about the importance of redox biomarkers in critically ill patients with neurosurgical and neurological conditions. Moreover, in clinical practice, redox biomarkers are not routinely measured to assess organ function or prognosis [33,34,35].

Acute traumatic brain injuries and acute neurological conditions constitute a significant medical problem affecting all age groups, and they are characterized by a serious and significant risk of neurological deficits. Imaging studies are currently the standard diagnostic tool. However, they demonstrate the morphology of brain damage and are essential for surgical decision making, but they do not predict functional dysregulation. Specific and sensitive biomarkers for brain tissue have not been described. Furthermore, it is also awaited to determine biomarkers of brain damage in material other than cerebrospinal fluid. In our study, we assessed markers of oxidative stress in various biological samples from patients with acute central nervous system disorders to examine their usefulness in predicting short-term prognosis and their correlation with other indicators of organ dysfunction.

The primary aim of this study was to explore the antioxidant and oxidant status in critically ill patients with brain injury. We decided to analyze antioxidant status using the most commonly assessed biomarkers—ferric reducing antioxidant power (FRAP) and TAC—and oxidative status using TOS assays. The secondary aim of the study was to investigate the correlation between well-known biomarkers of organ function and redox status of plasma, saliva, and urine; additionally, antioxidant and oxidant status were analyzed as prognostic factors.

We hypothesized that the critical state of brain injury is associated with antioxidant–oxidant imbalance. In addition, we tested the hypothesis that TAC, FRAP, and TOS in different biofluids provide clinical information about organ function and survival.

## 2. Materials and Methods

Approval for the study was obtained from the Bioethics Committee of the Medical University of Bialystok (APK.002.110.2024). The study was conducted in the period between March and September 2024, at the Department of Anaesthesiology and Intensive Therapy, University Hospital in Bialystok, Poland. The inclusion criterion was as follows: adult patients who qualified for intensive therapy due to an acute neurosurgical or neurological condition (traumatic brain injury, brain hematoma, ischemic stroke, hemorrhagic stroke, ruptured vascular aneurysm, or status epilepticus). Exclusion criteria included blood hemostasis dysregulation, autoimmunological disorders, end-stage metabolic disorders, diabetes mellitus, and septic shock.

The study group consisted of adult patients qualified for intensive therapy due to an acute serious neurosurgical (72% of the study group) or neurological (28% of the study group) condition. The typical medical procedures (central line cannulation, arterial cannulation, and mechanical ventilation with normocapnia and normoxia) and elements of pharmacology in intensive therapy (pharmacological coma, haemodynamic therapy to assess mean arterial pressure more than 90 mmHg, enteral and parenteral nutrition, osmotic therapy, gastric ulcus prophylaxis, and prophylaxis of thromboembolism) were conducted. The enteral nutrition was performed using Peptamen Intense (Nutricia), and parenteral nutrition mixtures were composed of SmofKabiven (Fresenius), Vitalipid (Fresenius), Optylite (Fresenius), Cernevit (Baxter), Supliven (Fresenius), Thiamina (Kwality Pharmaceuticals), 20% Magnesium Sulfate (Polpharma), 15% Kalium Chloratum (Fresenius). The details of the composition of the mixtures are available on the manufacturers’ websites.

This study was a non-randomized cohort study, and 50 patients were recruited in the first phase. Biological materials (blood, urine, and saliva) were collected according to the protocol on the first day after admission to the Intensive Care Unit.

Blood samples were aseptically drawn into tubes containing ethylenediaminetetraacetic acid (2.7 mL EDTA BD vacutainers) via an arterial line. Plasma was isolated after centrifugation (4000 rpm at 4 °C within 10 min of collection) and immediately stored at −80 °C in 300 μL aliquots in Eppendorf tubes for further analysis. Samples with signs of undesired hemolysis were excluded from the study. The supernatant fluid was preserved for the study and frozen at −80 °C until the determinations were made [23,31].

Saliva samples were collected from the oral cavity using a closed aspiration system. Due to specific intensive procedures, the saliva was collected in the morning, 4 h after stopping enteral nutrition, before cleaning and disinfection of the oral cavity. The supernatant obtained after centrifuging the samples was used for the study. Centrifugation was performed at 4 °C and 3000× *g* for 20 min. The resulting supernatant was stored at −80 °C [23,31,32].

Urine samples were collected from urinary catheters, centrifuged (4000 rpm at 4 °C within 10 min of collection), and immediately stored at −80 °C in 300 μL aliquots in Eppendorf tubes for further analysis (for no longer than 6 months).

To assess organ function, blood morphology, and plasma biochemical tests were performed, including for urea, creatinine, GFR—Glomerular Filtration Rate, alanine aminotransferase, aspartate aminotransferase, troponin, N-terminal pro-B-type natriuretic peptide (NT-proBNP), C-reactive protein, procalcitonin, arterial blood oxygen pressure (PaO_2_), lactates, albumins, and bilirubin. Information on vital functions, body temperature, catecholamine infusion, and mechanical ventilation parameters (FiO_2_) was recorded from medical documentation. APACHE II scores were calculated based on the obtained information and laboratory results.

The materials were immediately sent to the laboratory, where they were processed by specialists and stored until testing.

Due to complications encountered during laboratory testing (inability to collect samples, insufficient diagnostic material, and blood hemolysis), only the test results for 45 patients in the study group were analyzed.

The control group was recruited in the second stage in August of 2024. It consisted of 50 healthy volunteers, and materials were collected in the morning. The blood samples were assessed via venipuncture, urea was collected into aseptic probes, and saliva was collected into aseptic tubes before cleaning and disinfection of the oral cavity. After appropriate laboratory preparation, the materials (n = 49) were stored until testing.

### 2.1. Total Antioxidant Capacity

All reagents necessary for performing the redox assays were purchased from Sigma-Aldrich (Nümbrecht, Germany/Saint Louis, MO, USA). The absorbance of the samples was measured using a 96-well microplate reader (Biotek ELx800; BioTek Instruments, Winooski, VT, USA).

Total antioxidant capacity (TAC) was assessed using the colorimetric method described by Erel [1] using the ABTS (2,2-azinobis-3-ethylbenzothiazoline-6-sulfonic acid) radical cation, with Trolox (6-hydroxy-2,5,7,8-tetramethylchroman-2-carboxylic acid) serving as the standard. This method relies on assessing the capacity of the sample’s antioxidants to neutralize the 2,2-azino-bis (3-ethylbenzothiazoline-6-sulfonate) cation radical (ABTS+). Absorbance was measured spectrophotometrically at 660 nm [1]. The results are expressed as Trolox equivalents in µmol/L.

### 2.2. Total Oxidant Status

The total oxidant status (TOS) was determined using a bichromatic method (560/800 nm) based on the oxidation of Fe^2+^ to Fe^3+^ by oxidants present in the sample. The concentration of total oxidants was then calculated using a calibration curve based on hydrogen peroxide, as described by Erel [2]. Fe^3+^ was detected with xylenol orange [2]. The results are presented as H_2_O_2_ µmol Eq/L.

### 2.3. Oxidative Stress Index

Oxidative stress index (OSI) was determined as the ratio of TOS to TAC. The formula used for this calculation is OSI = TOS/TAC [2,3].

### 2.4. Ferric Reducing Antioxidant Power

The ferric reducing antioxidant power (FRAP) was assessed using a colorimetric method that involves the reduction of ferric ions (Fe^3+^) to ferrous ions (Fe^2+^) in an acidic environment. This reaction results in the formation of a colored ferrous tripyridyltriazine (Fe^2+^-TPTZ) complex whose absorbance was measured spectrophotometrically at 592 nm [4]. The results are expressed as Trolox equivalents in µmol/L.

### 2.5. Statistical Analysis

The numerical data are presented as the mean ± standard deviation, median, minimum–maximum range, 25–75th percentile range (IQR), or counts (n) with proportions (%). The Shapiro–Wilk test was used to assess the normality of the distributions of the quantitative variables. In a subsequent analysis, nonparametric tests were used to assess correlations between variables and groups. The Mann–Whitney U test was used to compare the study and control groups, as well as the survivors and non-survivors groups. The Wilcoxon paired *t*-test was used to compare the variables FRAP, TAC, and TOS across different biofluids. Spearman’s rank test was used to describe correlations between FRAP, TAC, TOC, OSI, and parameters of organ function. Spearman’s correlation coefficient was classified as follows: 0.00–0.19 “very weak”, 0.20–0.39 “weak”, 0.40–0.59 “moderate”, 0.60–0.79 “strong”, 0.80–1.0 “very strong”. Receiver operating characteristic (ROC) curves were used to assess the diagnostic utility of FRAP, TAC, TOS, and OSI. For each parameter, the area under the curve (AUC) and optimal cutoff values were determined to ensure high sensitivity and specificity. The study was a non-randomized cohort study, and 50 patients were recruited in the first phase. All calculations were performed using Statistica 14.0.0 (TIBCO Software Inc., Santa Clara, CA, USA), and *p* < 0.05 was used as the level of significance.

## 3. Results

### 3.1. General Characteristics

A total of 45 patients (17 women and 28 men) and 49 controls (23 women and 26 men) were included in the analysis. In the study group, 20 patients needed intensive therapy due to traumatic brain injury, 7 due to brain hematoma, 7 due to ischemic stroke, 6 due to hemorrhagic stroke, 3 due to ruptured brain aneurysm, and 2 patients due to status epilepticus. The groups did not differ in anthropometric parameters (age, weight, BSA, and BMI). The anthropometric data for the study and control groups are presented in Table 1.

Brain edema was diagnosed in 28 patients, intracranial hemorrhage in 18 patients, subarachnoid hemorrhage in 17 patients, intraventricular hemorrhage in 11 patients, and cortical–subcortical obliteration in 15 patients, based on CT scans. Epidural hematoma was noted in eight patients, skull fractures in seven patients, and signs of brain herniation in three patients. The overall mortality was 17.7% (8/45 cases), the 7-day mortality was 4.44% (2/45 cases), and the 14-day mortality was 8.88% (4/45 cases). The clinical parameters of organ function in the study group are presented in Table 2.

### 3.2. Redox Status

Statistically significant differences between the groups were observed for TAC in plasma, urine, and saliva (*p* < 0.05). The plasma and urinary TAC levels were 549.56 µmol/L and 999.03 µmol/L in the study group and 371.37 µmol/L and 1362.83 µmol/L in the control group. Additionally, the median salivary TAC concentration was 0.48 µmol/L in the study group, compared with 2.9 µmol/L in the control group.

Antioxidant properties, measured by the FRAP method, also differed between the groups. The median FRAP levels in plasma, urine, and saliva were 963.23 µmol/L, 79.31 µmol/L, and 470.99 µmol/L for the study group and 829.94 µmol/L, 157.33 µmol/L, and 1667.11 µmol/L for the control group (*p* < 0.05).

The plasma total oxidant status was significantly lower, with median concentrations of 6.32 µmol Eq/L H_2_O_2_ for the study group and 12.5 µmol Eq/L H_2_O_2_ for the control group (*p* < 0.05).

OSI levels for plasma and saliva were significantly different between the groups. In the study group, the OSI for plasma was 0.012 and for saliva was 14.81, while in the control group, the OSI level for plasma was 0.031 and for saliva was 3.86 (*p* < 0.001).

**Figure 1:**
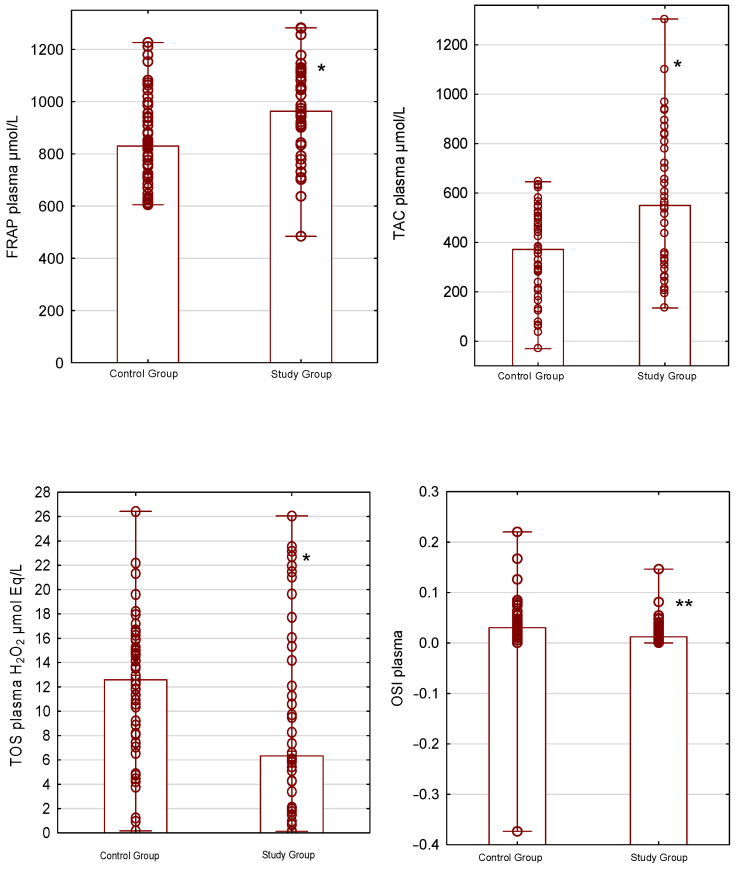
Plasma total antioxidant–oxidant status in the control and study groups. Box-and-whisker plots show ferric reducing ability of plasma (FRAP; μmol/L), total antioxidant capacity (TAC; μmol/L), total oxidant status (TOS; H_2_O_2_ μmol Eq/L), and the oxidative stress index (OSI). * *p* < 0.05, ** *p* < 0.001. Data are presented as bar chart with individual data points—median and minimum–maximum ranges.

Plasma total antioxidant–oxidant status based on plasma FRAP, TAC, TOS, and OSI in the control and study groups is presented in Figure 1.

**Figure 2:**
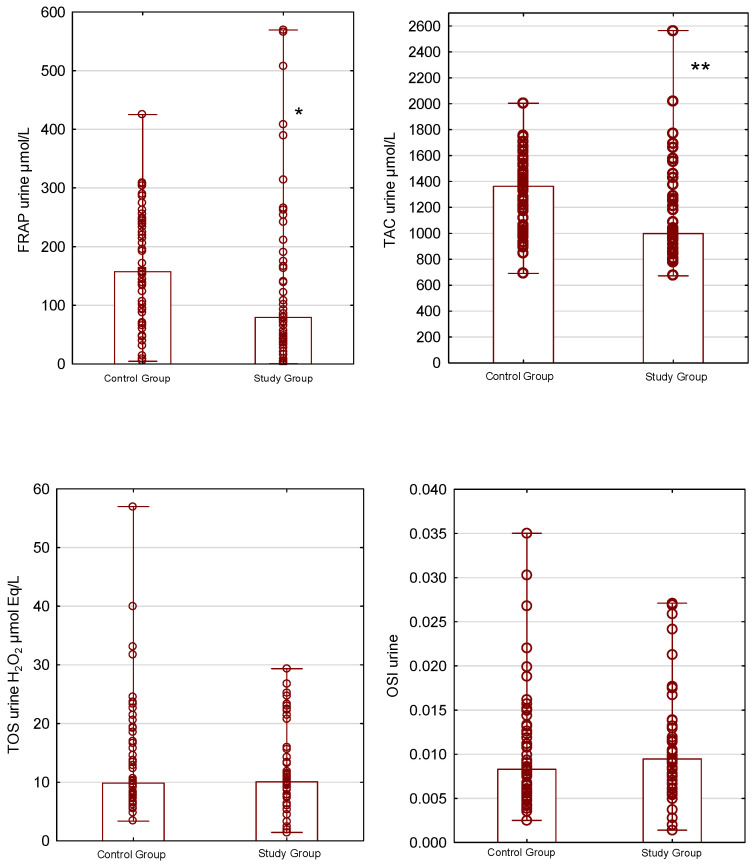
Urinary total antioxidant–oxidant status in the control and study groups. Box-and-whisker plots present ferric reducing ability of urine (FRAP; μmol/L), total antioxidant capacity (TAC; μmol/L), total oxidant status (TOS; H_2_O_2_ μmol Eq/L), and the urinary oxidative stress index (OSI). * *p* < 0.05, ** *p* < 0.001. Data are presented as bar chart with individual data points—median and minimum–maximum ranges.

Urinary total antioxidant–oxidant status on plasma FRAP, TAC, TOS, and OSI in the control and study groups is presented in Figure 2.

**Figure 3:**
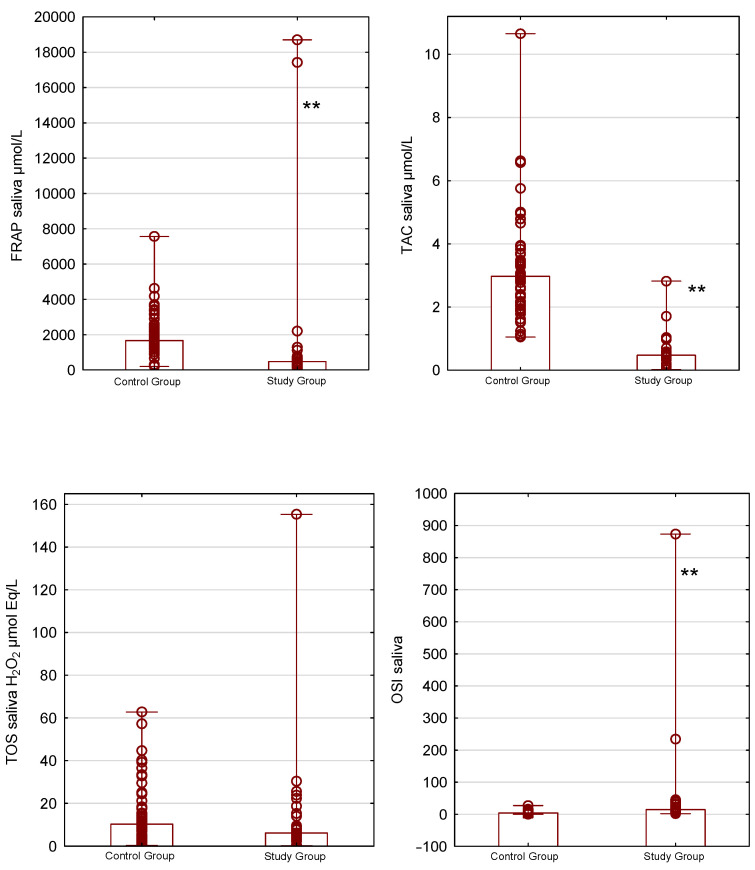
Salivary total antioxidant–oxidant status in the control and study groups. Box-and-whisker plots present ferric reducing ability of saliva (FRAP; μmol/L), total antioxidant capacity (TAC; μmol/L), total oxidant status (TOS; H_2_O_2_ μmol Eq/L), and the salivary oxidative stress index (OSI). ** *p* < 0.001. Data are presented as bar chart with individual data points—median and minimum–maximum ranges.

Salivary total antioxidant–oxidant status on plasma FRAP, TAC, TOS, and OSI in the control and study groups is presented in Figure 3.

Antioxidant activity based on plasma FRAP and plasma TAC had higher median values in the survivors group, whereas saliva FRAP and saliva TAC were higher in the non-survivors group. Moreover, median TOS values in plasma, urine, and saliva were higher in the non-survivors group. A comparison of median FRAP, TAC, TOS, and OSI in survivors and non-survivors revealed no statistically significant differences (Table 3).

**Figure 4:**
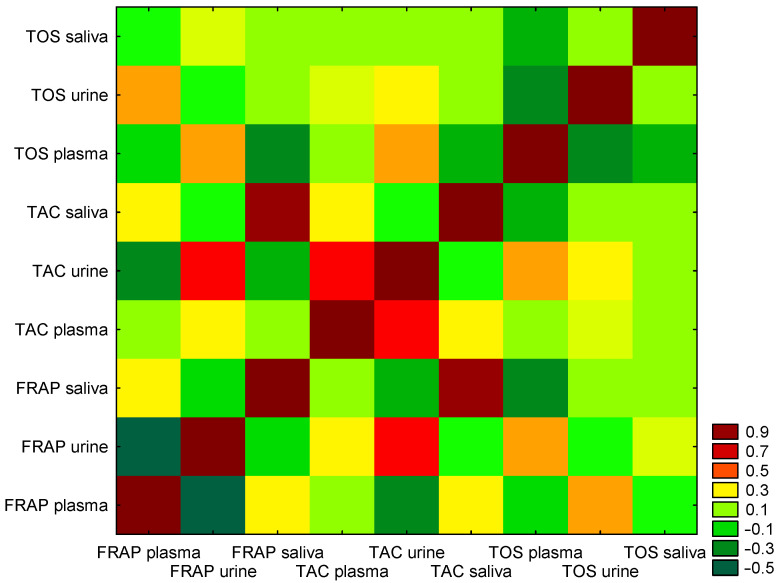
Heat map of correlations between redox status of FRAP, TAC, and TOS in plasma, urine, and saliva in study group.

Spearman’s test revealed significant correlations between redox status in plasma, urine, and saliva in the study group. A weak positive correlation was noted between antioxidant activity expressed as plasma FRAP and plasma TAC (r = 0.22, *p* < 0.05) and urinary FRAP and urinary TAC (r = 0.36, *p* < 0.05). A very strong positive correlation was noted between salivary FRAP and salivary TAC (r = 0.93, *p* < 0.05). A moderate correlation was observed between salivary FRAP and salivary TOS (r = 0.47, *p* < 0.05). Detailed results of Spearman’s correlation analysis are presented in Table 4. The heat map of correlations between redox status of FRAP, TAC, and TOS in plasma, urine, and saliva in the study group is presented in Figure 4.

Additionally, significant correlations were observed between the salivary FRAP and salivary TAC and creatinine (r = −0.9, *p* = 0.04, and r = −0.9, respectively) in the non-survivors of the study group. We did not find any correlations between plasma antioxidative biomarkers and urea or creatinine. The salivary TAC and plasma TOS levels correlated with urea (r = −0.90, *p* = 0.05; r = −0.82, *p* = 0.05). The eGFR correlated with TAC urine and TAC saliva, rho 0.76, *p* = 0.03, and rho 0.90, *p* = 0.02, and TOS plasma with rho 0.94, *p* = 0.03. Plasma troponin levels were associated with salivary FRAP (r = −0.90, *p* = 0.03) and urinary TAC levels (r = −0.85, *p* = 0.04). The oxygenation ratio, defined as the P_a_O_2_/FiO_2_ ratio, was correlated with plasma FRAP (r = −0.94, *p* = 0.04) and urinary TAC (r = −0.78, *p* = 0.02). The CRP level was correlated with the salivary FRAP (r = −0.90, *p* = 0.02), while PCT was associated with plasma TAC (r = 0.82, *p* = 0.04). Oxidative status, as measured by urinary TOS, correlated with lactate levels (r = −0.83, *p* = 0.03). The eGFR correlated with saliva TOS in the survivor group with rho 0.54, *p* = 0.4. The analysis did not show a correlation between redox status and severity state, as measured by the APACHE II score, in survivors and non-survivors of the study group. The results of Spearman’s correlation analysis are presented in Table 5 and Table 6.

**Figure 5:**
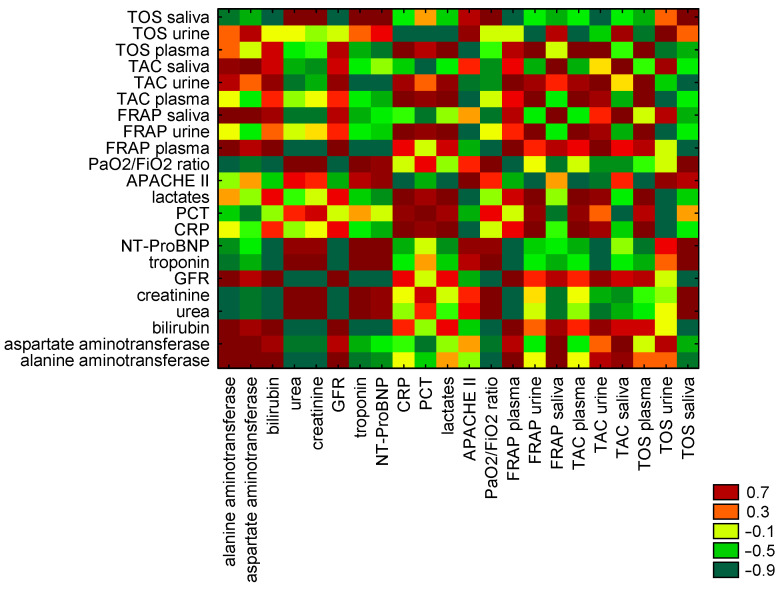
Heat map of correlations between redox status of FRAP, TAC, and TOS in plasma, urine, and saliva, and biomarkers of organ function in non-survivors of study group.

**Figure 6:**
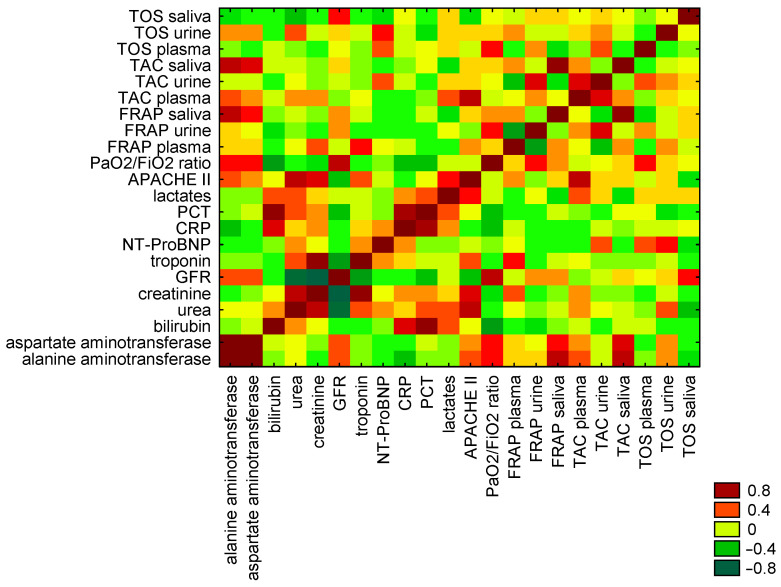
Heat map of correlations between redox status of FRAP, TAC, and TOS in plasma, urine, and saliva, and biomarkers of organ function in survivors of study group.

The heat maps of correlations between redox status of FRAP, TAC, and TOS in plasma, urine, and saliva, and biomarkers of organ function in non-survivors and survivors of study group are presented in Figure 5 and Figure 6.

**Figure 7:**
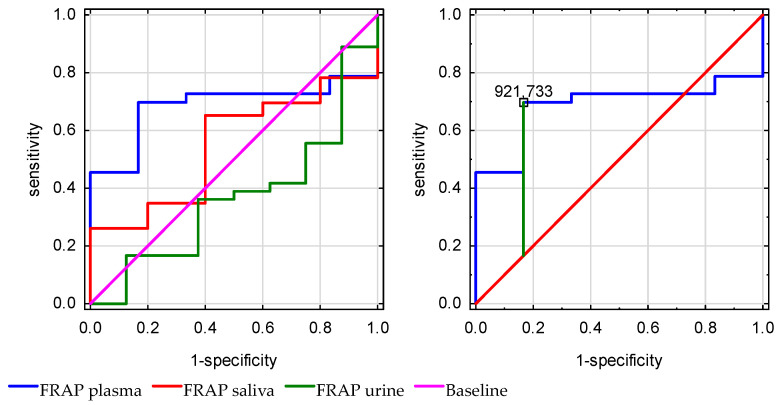
Receiver operating characteristic (ROC) curves of plasma, urine, and saliva ferric reducing antioxidant power (FRAP) for the prediction of in-hospital mortality.

For overall in-hospital mortality analysis, plasma FRAP discriminated significantly better (AUC = 0.687, 95% CI: 0.521–0.853, *p* = 0.02) compared with urine (*p* = 0.21, AUC = 0.36) and salivary FRAP (*p* = 0.69, AUC = 0.54). A plasma FRAP level below 921.733 μmol/L was significantly associated with mortality, with 69% sensitivity and 83% specificity (Figure 7).

**Figure 8:**
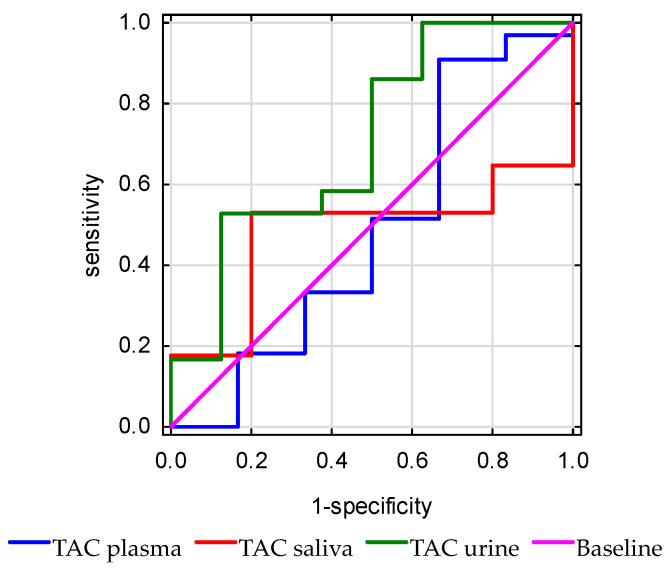
Receiver operating characteristic (ROC) curves of total antioxidant capacity (TAC) measured in plasma, urine, and saliva for the prediction of in-hospital mortality.

The plasma TAC (*p* = 0.92) did not discriminate mortality significantly better than urine (*p* = 0.88), and saliva (*p* = 0.056) (Figure 8).

**Figure 9:**
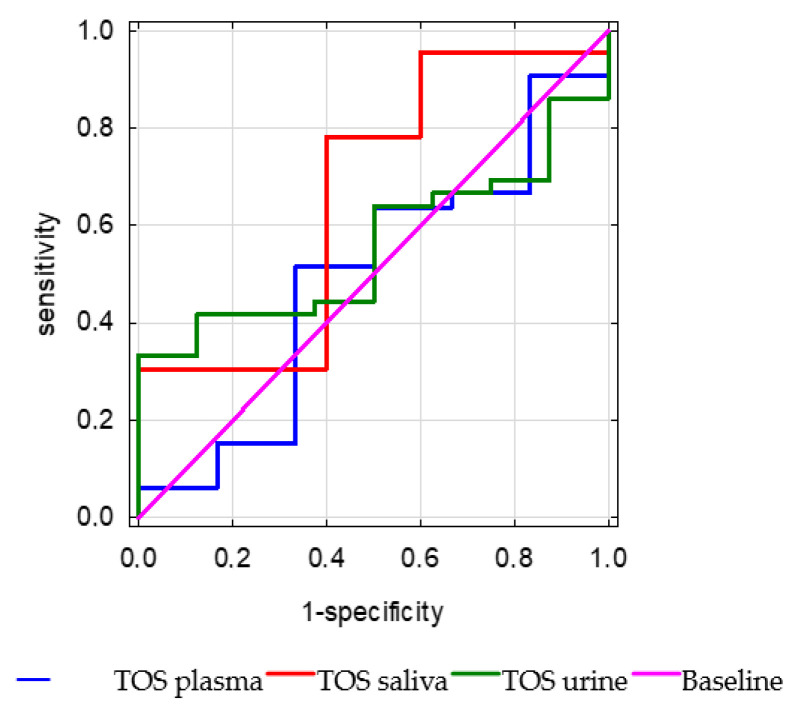
Receiver operating characteristic (ROC) curves of total oxidant status (TOS) measured in plasma, urine, and saliva for the prediction of in-hospital mortality.

The plasma TOS (*p* = 0.93, AUC = 0.49) did not discriminate significantly better mortality than urine (*p* = 0.51, AUC = 0.55) or saliva (*p* = 0.27, AUC = 0.66) (Figure 9).

**Figure 10:**
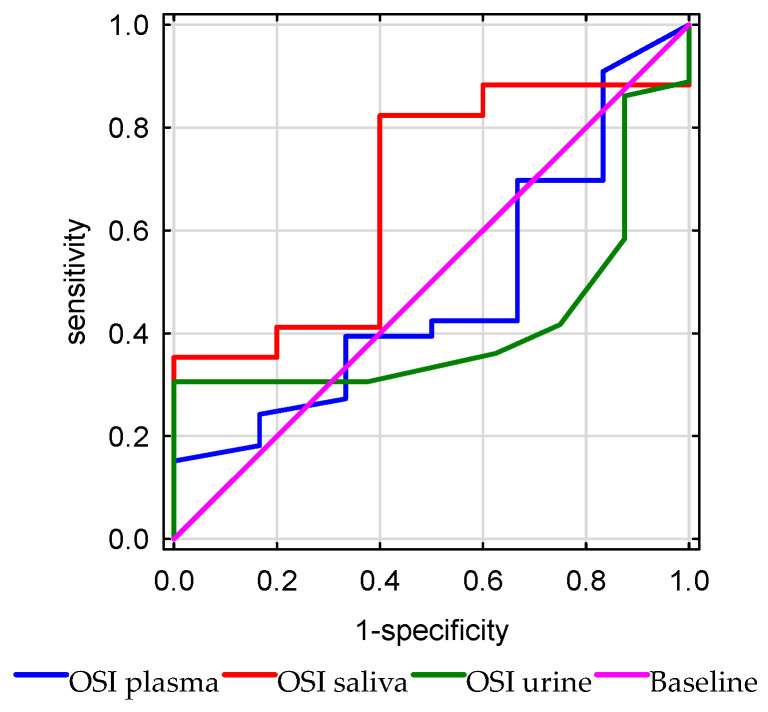
Receiver operating characteristic (ROC) curves of the oxidative stress index (OSI) measured in plasma, urine, and saliva for the prediction of in-hospital mortality.

The plasma, urinary, and salivary OSI did not discriminate general in-hospital mortality (*p* = 0.88, AUC = 0.48), (*p* = 0.38, AUC = 0.41), (*p* = 0.23, AUC = 0.67), respectively (Figure 10).

## 4. Discussion

Our study showed significant differences in redox homeostasis across various biological fluids in critically ill patients following neurosurgery. The antioxidant status, as indicated by plasma FRAP and TAC, was significantly higher in the study group compared to healthy controls. On the other hand, FRAP and TAC levels were considerably lower in urine and saliva. We did not find any correlation between central (plasma) and local (urine, saliva) redox homeostasis. There was no significant difference between survivors and non-survivors.

In traumatic brain injury, oxidative stress affects neurons and supporting cells (astrocytes) due to direct mechanical damage and impaired cerebral circulation [17,18,19,20]. Brain tissue is highly susceptible to the harmful effects of ROS due to its high oxygen consumption, low antioxidant capacity, and limited regenerative mechanisms. Lipid peroxidation plays a vital role in brain injury, leading to cell membrane lysis, disrupting ion balance and mitochondrial function, and inducing apoptosis. As a result of blood–brain barrier disruption, redox biomarkers can enter the bloodstream and other biological fluids [16,17,18,19,20,21].

The literature lacks data on the relationship between blood, urine, and saliva redox homeostasis in critically ill patients with brain injury and secondary organ failure. Our research is the first to measure the antioxidant and oxidative status of various biological fluids. In the study group, we have demonstrated an increase in blood antioxidant defense mechanisms (increasing TAC and FRAP) and protection against systemic oxidative stress (decreasing TOS and OSI). Although oxidative stress is inextricably linked to traumatic brain injury, an increase in blood TAC and FRAP and a decrease in blood TOS and OSI may result from anesthetic management of critically ill patients [33,34,35,36,37,38]. The principles of intensive therapy for patients with brain damage include neuroprotective pharmacological sedation, intubation and controlled mechanical ventilation, fluid management and hemodynamic stabilization, antithrombotic prophylaxis, and nutritional support [30,39,40,41,42,43,44]. The pharmacological coma is induced by continuous intravenous infusion of anesthetics (propofol or thiopental), benzodiazepines (midazolam or dexmedetomidine), and opioids (fentanyl and derivatives). Many of these substances may exhibit antioxidant effects by inhibiting neuronal transmission (NMDA and calcium channels transmission) and reducing pro-oxidant enzymes. The antioxidant potential of blood can also be enhanced by enteral and parenteral nutritional therapy, including immunomodulatory amino acids such as glutamate and arginine, unsaturated fatty acids, micro- and macronutrients, and vitamins [6,12,14,17,45,46,47]. In our study, nutritional treatment was provided using an industrial enteral diet and pharmacy parenteral mixtures without targeted and additional antioxidant supplementation. The neuroprotective pharmacological coma was conducted according to the recommendations of neurocritical care standards with intravenous anaesthetics, benzodiazepines, and opioids.

The use of circulating redox biomarkers is increasingly being criticized. Redox homeostasis is constantly changing and can be limited only to the local cells or tissues where the disease process occurs. In our study, we found no correlation between central (plasma) and local (urine and saliva) redox homeostasis, suggesting compartment-dependent oxidative stress. Unlike blood, the antioxidant potential of urine and saliva was significantly lower in the study group (decreasing FRAP and TAC), whereas TOS did not differ significantly. This indicates local depletion of antioxidant mechanisms and increased susceptibility to oxidative damage. In our study, none of the patients met the criteria for acute secondary kidney injury and qualification for renal replacement therapy (mean creatinine concentration was 0.85, median 0.73, maximum value 2.24, mean, median eGFR was 101). In non-survivors, we observed strong negative relationships between salivary TAC and creatinine and urea (r = −0.9), and between salivary FRAP and creatinine (r = −0.9). However, we found a strong correlation between urine and saliva antioxidant status and renal function measured by eGFR. This should come as no surprise, as saliva content largely depends on kidney function and electrolyte composition. Although further research is needed, measuring TAC and FRAP in the saliva of critically ill patients may be a better indicator of renal redox homeostasis than central oxidative stress [16,17,22,23,28].

The reduced TAC and FRAP in the saliva and urine of critically ill patients with brain injury may also result from the specific nature of the analytical method and biological fluid. TAC measures the concentration of non-enzymatic antioxidants using 2,2′-azino-bis(3-ethylbenzothiazoline-6-sulfonic acid) (ABTS), while FRAP assesses the reduction of iron ions (Fe^3+^) to iron ions (Fe^2+^). These methods differ in the contributions of individual antioxidants to total antioxidant activity; e.g., the proportion of uric acid in TAC is much lower than in FRAP, and bilirubin has no effect on FRAP content. In contrast to plasma, the antioxidant potential of urine and saliva depends largely on uric acid and, to a lesser extent, on protein thiol groups, bilirubin, vitamin C, and polyphenols. Therefore, it is recommended to assess redox homeostasis using several methods simultaneously [29,30,48,49,50].

We found no differences in the antioxidant–oxidative status of plasma, urine, and saliva between survivors and non-survivors. Although the lack of differences may be due to the small size of the study group (and subgroups), it may also result from the treatment used or the limited diagnostic usefulness of redox biomarkers.

In our study, the overall mortality was 17.7%, with a 7-day mortality of 4.44% and a 14-day mortality of 8.88%. A similar mortality rate was presented in Collaborative European NeuroTrauma Effectiveness Research in TBI, while in severe brain trauma, mortality increases to 20%, but the long-term survival rate was about 90% for the 30 and 90-day survival. The leading cause of death was primary brain injury in 64%, but the secondary organ dysfunction was in 24% of patients [29,35,48,49,50,51].

We have demonstrated that plasma FRAP concentrations below 921.733 µmol/L were associated with increased mortality, with a sensitivity of 69% and a specificity of 83%. Antioxidant–oxidative status was compared with other parameters of physical (APACHE II) and neurological state (Glasgow Coma Scale), and radiological scales (Marshall scale, Rotterdam scale) as fundamental elements of mortality predicting models. Therefore, serum TAC concentrations above 450 µmol/L had a sensitivity of 72% and a specificity of 52% for a favorable prognosis in brain injuries (AUC = 0.662, *p* < 0.0001). Additionally, serum TAC was significantly lower in survivors (median = 2.38 mmol/mL than in non-survivors (median = 5.33 mmol/mL). The AUC of 0.82 for serum TAC indicated the good predictive value of 30-day mortality (*p* < 0.001). Patients with serum TAC higher than 3.39 mmol/mL had a higher 30-day mortality (Hazard Ratio = 4.5, *p* < 0.001). Moreover, serum TAC values were associated with 30-day mortality after adjusting for GCS and age (OR = 1.92, *p* = 0.006). Serum TAC levels of more than 3.39 nmol/mL predicted 30-day mortality with a sensitivity of 72%, a specificity of 79%, a positive predictive value of 75%, and a negative predictive value of 73% [37].

In the other study, serum TAC higher than 2.59 nmol/mL was associated with 30-day mortality after controlling for APACHE-II and Rotterdam Scale (OR = 4.40; *p* = 0.03), also controlling for GCS and age (OR = 5.88; *p* = 0.009), and controlling for Rotterdam Scale and abnormal pupils at admission (OR = 3.89; *p* = 0.02). The overall predictive capacities of the first, second, and third models for 30-day mortality were 85.4%, 82.3%, and 76.0%, with AUC values of 0.79, 0.74, and 0.59, respectively [39]. The non-surviving patients had significantly higher serum TAC concentrations on the first, fourth, and eighth days compared with surviving patients. Moreover, authors presented a statistical association between serum TAC levels and 30-day mortality, after controlling for platelet count, GCS, and lactic acid (OR = 2.672; *p* = 0.001), as well as for sex, glycemia, and age (OR 1.969; *p* = 0.002) [40,41]. Studies conducted in patients after subarachnoid hemorrhage also showed that TAC correlated with worse neurological prognosis at the 6-week and 6-month assessments [42,43].

We did not observe any correlation between mortality and redox biomarkers in saliva and urine. Further studies should verify whether urinary and salivary TAC, TOS, and OSI reflect only local changes in redox homeostasis or indicate systemic shifts.

Sufficient scientific research evaluating the correlation between oxidative stress and secondary organ function in patients requiring intensive care is still lacking. Our study found a negative correlation between saliva TAC and the concentration of urea and creatinine in non-survivors. Moreover, correlation with eGFR and TAC urine and TAC saliva was described. Plasma troponin values were negatively correlated with antioxidant biomarkers in plasma and urine, as determined using the FRAP method. A negative correlation was also found between the concentration of NT-proBNPa and plasma OSI. Oxygenation index was correlated with plasma FRAP (Rho = −0.94) and urine TAC (Rho = −0.78). CRP was negatively correlated with salivary FRAP, while procalcitonin showed a positive correlation with plasma TAC. In our study, we demonstrated a correlation between antioxidant potential and parameters of renal and cardiac function, and inflammation in a group of critically ill neurological and neurosurgical patients. In these medical situations, the observed organ dysfunctions are secondary.

We did not find any similar studies in the available literature. However, the interrelationships between biomarkers of oxidative stress in primary organ failures, including heart disease and renal failure, are well understood. The mechanisms of secondary organ damage in acute neurological and neurosurgical conditions are well described and influence the mortality. An acute lung injury (ALI) is the most frequent secondary dysfunction following acute brain injury, and it is estimated to be 22%, with mortality ranging between 28 and 33%. The mortality rate is significantly higher in patients with severe brain trauma with ALI than in non-ALI patients (38% vs. 15%). Secondary acute lung injury and poor neurological score based on the Glasgow Coma Scale were also associated with higher short-term mortality. Therefore, the other symptoms of neurological injury described in intracranial abnormalities in CT scans did not correspond with the risk of development of secondary ALI and in-hospital mortality. Moreover, other secondary systemic dysregulations like respiratory failure, heart failure, arrhythmias, and arterial hypotension (arterial systolic pressure < 90 mm Hg) also did not correlate with short-term mortality in patients with brain injury [52,53,54,55].

We found that measuring the antioxidative status of plasma, urine, and saliva in patients with acute brain damage using TAC and FRAP methods gave similar results. The highest median FRAP and TAC values in the Study Group were found in plasma, followed by urine and then saliva. Urine and saliva measurements were correlated with biomarkers of organ functions. Therefore, we conclude that non-invasive and simple urine and saliva collection and analyses may be the future of the laboratory diagnosis of oxidative stress in patients with brain trauma.

It should be remembered that age, gender, metabolic disorders (diabetes and obesity), eating habits (diet), and lifestyle factors (caffeine and alcohol) are crucial for assessing redox homeostasis [50,56,57,58,59,60,61]. Therefore, in our study, we recruited individuals without comorbidities with oxidative stress etiology. The control group consisted of individuals of similar age and gender to the study group. However, these confounding variables cannot be completely ruled out.

The main limitations of this study are the small number of patients and the short recruitment period. Second, we did not have information on the initial neurological states of the patients; patients admitted to the ICU were under pharmacological sedation, and our analysis of neurological outcomes was limited to survivors and non-survivors. Third, biological samples were collected only once, making it difficult to conclude the prognostic value of redox biomarkers. Therefore, it is recommended to increase the number of patients in future studies, conduct a long-term assessment of redox homeostasis dynamics, and include additional parameters (e.g., the impact of specific pharmacotherapy and nutritional treatment) in the analyzed model. The aim of this study was to describe the redox balance using FRAP, TAC, and TOC in various biological samples and compare them with parameters of organ function in patients with brain damage. The study protocol was very short, with biological samples collected only once, on the first 24 h of hospitalization. However, a long-term follow-up study of clinical observation and comparing the redox status with neurological conditions and long-term prognosis will be an interesting continuation. It should be noted that reference values have not yet been established for the parameters measured, not only for saliva, but also for urine and blood. In the literature, TAC and TOS levels in healthy individuals vary significantly depending on the study [18,62]. These differences may be due to the analytical method, reaction conditions, sample preparation, and storage, as well as numerous individual factors.

Summarizing, our research indicates differences in central (plasma) and local (urine, saliva) redox homeostasis in critically ill patients with acute brain injury and secondary organ failure. The antioxidant and oxidant status did not differ between survivors and non-survivors; however, circulating redox biomarkers strongly correlated with organ function in non-survivors. Our study is the first to describe correlations between oxidative stress parameters (TAC, TOS, TOS/TAC) and laboratory parameters assessing organ function in patients with brain injury in the intensive care unit using different biofluids. Although further research is needed, measuring TAC and FRAP in the saliva of critically ill patients may be a better indicator of local redox balance than central oxidative stress.

Sufficient scientific research evaluating the correlation between oxidative stress and secondary organ function in patients with brain injury requiring intensive care is still lacking. Therefore, the results of the study enrich the limited knowledge in oxidative stress measurements in different biofluids in critically ill patients with acute neurological and neurosurgical disorders. Due to the serious health consequences of these conditions, studies for biomarkers of short-term as well as long-term prognosis are necessary. Moreover, interesting and promising advancements in intensive care for acute brain injury would be the use of oxidative stress assays in the prognosis of critically ill patients and the modification of therapy aimed at enhancing antioxidant capacity and limiting oxidative stress mechanisms. Furthermore, development potential is associated with the use of various biological materials, especially those obtained noninvasively, easily, and readily available for laboratory testing, including oxidative stress parameters.

## Figures and Tables

**Table 1:** Anthropometric data of the study and control groups. BMI—body mass index. The mean, standard deviation (SD), median, minimum–maximum ranges, and interquartile ranges (IQR 25–IQR 75) are presented.

		Mean	SD	Median	Minimum–Maximum	IQR 25–IQR 75
Age (years)	Control group	59.72	9.25	59.50	44.00–81.00	52.00–66.00
	Study group	61.30	15.24	65.00	23.00–84.00	46.00–74.00
Weight (kg)	Control group	84.80	17.36	84.00	56.00–120.00	77.00–95.00
	Study group	79.44	19.31	74.00	52.00–125.00	65.00–92.00
Height (cm)	Control group	173.33	10.34	173.00	150.00–196.00	164.00–181.00
	Study group	170.42	8.90	170.00	152.00–188.00	166.00–175.00
BMI	Control group	28.44	4.21	28.39	16.83–42.51	25.60–31.23
	Study group	27.34	6.53	25.60	17.55–45.36	23.01–30.69

**Table 2:** Clinical characteristics of the study group.

	Mean	SD	Median	Minimum–Maximum	IQR 25–IQR 75
Hospitalization (days)	25.40	17.57	19.00	4.00–58.00	10.00–34.00
Alanine aminotransferase (U/L)	60.95	98.96	30.00	7.00–602.00	19.00–56.00
Aspartate aminotransferase (U/L)	65.53	103.79	40.00	14.00–704.00	25.00–70.00
Bilirubin (mg/dL)	0.69	0.46	0.63	0.20–2.37	0.35–0.76
Urea (mg/dL)	71.61	44.43	68.48	17.12–218.82	40.66–87.74
Creatinine (mg/dL)	0.85	0.41	0.73	0.35–2.24	0.57–1.16
GFR (mL/min)	110.22	52.92	101.00	30.00–243.00	66.00–146.00
Troponin (ng/L)	1051.20	6178.31	16.70	10.00–41,373.80	10.00–44.40
NT–proBNP (pg/mL)	1675.01	2695.49	333.90	21.20–10,423.30	116.10–1223.00
C–reactive protein (mg/L)	117.78	86.89	108.40	8.80–341.70	55.00–178.50
Procalcitoin (ng/mL)	1.11	3.12	0.23	0.03–19.54	0.11–0.58
Lactates (mmol/L)	1.15	0.50	1.00	0.46–3.08	0.88–1.31
APACHE II score	23.66	4.22	23.00	16.00–36.00	21.00–25.00
P_a_O_2_/FiO_2_ ratio	280.78	171.49	24.75	28.00–933.60	167.80–343.66

**Table 3:** Comparison of plasma, urinary, and salivary total antioxidant–oxidant parameters between survivors and non-survivors. The median, minimum–maximum values, interquartile range (IQR), and *p*-values are presented. FRAP—ferric reducing antioxidant power; TAC—total antioxidant capacity; TOS—total oxidant status; OSI—oxidative stress index.

	Non-Survivors (n = 8)	Survivors (n = 37)	*p*
FRAP plasma (µmol/L)	986.63 (484.09–1282.69; 834.18–1123.58)	904.43 (779.26–1044.75; 842.75–912.65)	*p* > 0.05
FRAP urine (µmol/L)	67.94 (0.15–566.57; 37.39–171.22)	111.92 (8.06–569.38; 71.23–247.69)	*p* > 0.05
FRAP saliva (µmol/L)	492.42 (7.57–18,700.50; 293.01–762.56)	404.32 (219.04–732.94; 306.14–676.72)	*p* > 0.05
TAC plasma (µmol/L)	534.06 (134.2–1100.49; 310.52–809.24)	616.78 (193.91–1304.43; 244.31–870.34)	*p* > 0.05
TAC urine (µmol/L)	1022.90 (794.72–2014.33; 883.88–1276.20)	918.56 (672.45–1457.54; 727.32–1010.43)	*p* > 0.05
TAC saliva (µmol/L)	0.53 (0.01–2.82; 0.17–0.979)	0.47 (0.20–1.03; 0.47–0.48)	*p* > 0.05
TOS plasma (H_2_O_2_ µmol Eq/L)	6.58 (0.13–26.06; 1.75–14.18)	5.18 (0.17–23.16; 3.38–21.03)	*p* > 0.05
TOS urine (H_2_O_2_ µmol Eq/L)	10.06 (1.45–26.74; 7.44–18.39)	9.85 (5.39–13.39; 8.32–11.37)	*p* > 0.05
TOS saliva (H_2_O_2_ µmol Eq/L)	6.14 (0.20–155.31; 4.08–18.67)	3.45 (0.60–13.94; 2.38–9.47)	*p* > 0.05
OSI plasma	0.011 (0.0002–0.15; 0.0033–0.031)	0.01 (0.0002–0.041; 0.0049–0.031)	*p* > 0.05
OSI urine	0.009 (0.0014–0.003; 0.0064–0.014)	0.01 (0.005–0.013; 0.0097–0.013)	*p* > 0.05
OSI saliva	14.91 (1.93–873.40; 8.9–36.87)	4.92 (2.9–29.59; 3.34–19.93)	*p* > 0.05

**Table 4:** Spearman’s correlation coefficients (r) between total antioxidant–oxidant parameters assessed in plasma, urine, and saliva. FRAP—ferric reducing antioxidant power; TAC—total antioxidant capacity; TOS—total oxidant status. Statistically significant correlations are considered at *p* < 0.05.

		FRAP			TAC			TOS		
		Plasma	Urine	Saliva	Plasma	Urine	Saliva	Plasma	Urine	Saliva
FRAP	Plasma	1.00	−0.21	−0.01	0.22	−0.17	−0.05	−0.06	−0.01	−0.05
	Urine	−0.21	1.00	0.13	0.08	0.36	0.18	0.08	0.11	0.14
	Saliva	−0.01	0.13	1.00	0.06	0.09	0.93	0.09	0.01	0.47
TAC	Plasma	0.22	0.08	0.06	1.00	0.08	0.10	0.03	0.09	0.25
	Urine	−0.17	0.36	0.09	0.08	1.00	0.25	0.17	0.40	0.12
	Saliva	−0.05	0.18	0.93	0.10	0.25	1.00	0.17	0.11	0.36
TOS	Plasma	−0.06	0.08	0.09	0.03	0.17	0.17	1.00	−0.01	−0.08
	Urine	−0.01	0.11	0.01	0.09	0.40	0.11	−0.01	1.00	−0.12
	Saliva	−0.05	0.14	0.47	0.25	0.12	0.36	−0.08	−0.12	1.00

**Table 5:** Spearman’s correlation coefficients (r) between total antioxidant–oxidant parameters (FRAP, TAC, and TOS in plasma, urine, and saliva) and selected biochemical and clinical variables in non-survivors. FRAP—ferric reducing antioxidant power; TAC—total antioxidant capacity; TOS—total oxidant status; NT-proBNP—N-terminal pro-B-type natriuretic peptide; APACHE II—Acute Physiology and Chronic Health Evaluation II; PaO_2_/FiO_2_ ratio—ratio of arterial oxygen partial pressure to fraction of inspired oxygen (oxygenation index), GFR—Glomerular Filtration Rate.

Non-Survivors	FRAP			TAC			TOS		
	Plasma	Urine	Saliva	Plasma	Urine	Saliva	Plasma	Urine	Saliva
Alanine aminotransferase	−0.25	−0.28	0.20	−0.48	−0.26	0.70	−0.08	−0.28	−0.60
Aspartate aminotransferase	−0.14	−0.04	0.02	−0.37	−0.41	0.40	0.02	−0.25	−0.70
Bilirubin	0.05	0.01	−0.10	0.55	−0.03	0.60	0.75	−0.46	−0.50
Urea	0.02	0.07	−0.60	0.14	−0.33	−0.90	−0.82	0.16	0.30
Creatinine	−0.25	0.26	−0.90	0.02	−0.69	−0.90	−0.77	−0.33	−0.30
GFR	0.42	0.04	0.60	0.37	0.76	0.90	0.94	0.02	0.00
Troponin	−0.14	−0.02	−0.90	−0.25	−0.85	−0.60	−1.00	−0.23	−0.30
NT-proBNP	−0.14	−0.52	−0.30	0.42	−0.28	0.20	−0.65	−0.16	0.40
C-reactive protein	0.42	0.54	−0.90	1.00	−0.38	−0.50	0.25	−0.47	−0.70
Procalcitonin	0.31	0.09	−0.50	0.82	0.02	−0.30	−0.20	−0.35	−0.10
Lactates	−0.65	0.02	−0.70	−0.20	−0.61	−0.20	−0.14	−0.83	−0.60
APACHE II	0.05	−0.20	0.21	0.78	0.20	−0.36	0.05	0.25	0.79
P_a_O_2_/FiO_2_ ratio	−0.94	0.09	−1.00	−0.31	−0.78	−0.70	−0.08	−0.66	−0.40

**Table 6:** Spearman’s correlation coefficients (r) between total antioxidant–oxidant parameters: FRAP, TAC, and TOS in plasma, urine, and saliva, and selected biochemical and clinical variables in survivors. APACHE II—Acute Physiology and Chronic Health Evaluation II; FRAP—ferric reducing antioxidant power; NT-proBNP—N-terminal pro-B-type natriuretic peptide; PaO_2_/FiO_2_ ratio—ratio of arterial oxygen partial pressure to fraction of inspired oxygen (oxygenation index); TAC—total antioxidant capacity; TOS—total oxidant status, GFR—Glomerular Filtration Rate.

Survivors	FRAP			TAC			TOS		
	Plasma	Urine	Saliva	Plasma	Urine	Saliva	Plasma	Urine	Saliva
Alanine aminotransferase	−0.06	−0.02	−0.28	0.32	0.24	0.36	−0.03	0.34	−0.39
Aspartate aminotransferase	−0.08	−0.12	−0.33	0.28	0.22	0.29	−0.02	0.28	−0.40
Bilirubin	−0.11	−0.24	−0.27	−0.07	−0.12	−0.04	−0.05	−0.07	−0.37
Urea	−0.01	−0.11	−0.26	0.06	−0.07	0.06	−0.02	−0.06	−0.63
Creatinine	0.06	−0.24	−0.11	0.14	−0.15	0.24	0.11	−0.26	−0.30
GFR	−0.11	0.21	0.19	0.00	0.19	−0.26	−0.11	0.23	0.54
Troponin	0.51	−0.18	0.03	0.23	−0.17	0.07	0.08	−0.08	−0.52
NT-proBNP	0.48	−0.01	−0.19	−0.13	−0.07	−0.20	0.05	−0.05	−0.56
C-reactive protein	0.05	−0.29	0.04	−0.20	−0.17	−0.19	−0.09	−0.20	0.26
Procalcitonin	0.28	−0.24	−0.09	0.02	−0.21	0.09	−0.06	−0.12	−0.29
Lactates	−0.07	−0.14	−0.38	0.02	0.02	−0.21	0.06	−0.02	−0.31
APACHE II	0.27	−0.08	0.15	−0.01	0.03	0.17	−0.02	0.04	−0.22
P_a_O_2_/FiO_2_ ratio	−0.11	0.14	0.33	0.16	−0.04	−0.06	−0.12	0.05	0.35

## Data Availability

The data presented in this study are available on request from the corresponding author. Data available on request due to privacy restrictions or ethical reasons.

## References

[1] Reed E., Case A. (2023). Defining the nuanced nature of redox biology in post-traumatic stress disorder. Front. Physiol..

[2] Cobley J., Margaritelis N., Chatzinikolaou P., Nikolaidis M., Davison G. (2024). Ten “Cheat Codes” for Measuring Oxidative Stress in Humans. Antioxidants.

[3] Breitenbach M., Eckl P. (2015). Introduction to Oxidative Stress in Biomedical and Biological Research. Biomolecules.

[4] Lemineur T., Deby-Dupont G., Preister J. (2006). Biomarkers of oxidative stress in critically ill patients: What should be measured, when and how?. Curr. Opin. Clin. Nutr. Matab. Care.

[5] Sies H. (2021). Oxidative eustress: On constant alert for redox homeostasis. Redox Biol..

[6] Marrocco I., Altieri F., Peluso I. (2017). Measurements and clinical significance of biomarkers of oxidative stress in Humans. Oxidative Med. Cell. Longev..

[7] Kościuczuk U., Jakubów P., Tarnowska K., Rynkiewicz-Szczepańska E. (2024). Opioid therapy and implications for oxidative balance: A clinical study of total oxidative capacity (TOC) and total antioxidative capacity (TAC). J. Clin. Med..

[8] Biedrzycki G., Wolszczak-Biedrzycka B., Dorf J., Maciejczyk M. (2024). The antioxidant barrier, oxidative/nitrosative stress, and protein glycation in allergy: From basic research to clinical practice. Front. Immunol..

[9] Więdłocha M., Zborowska N., Marcinowicz P., Dębowska W., Dębowska M., Zalewska A., Maciejczyk M., Waszkiewicz N., Szulc A. (2023). Oxidative Stress Biomarkers among Schizophrenia Inpatients. Brain Sci..

[10] Jorgensen A., Baago I.B., Rygner Z., Jorgensen M.B., Andersen P.K., Kessing L.V., Poulsen H.E. (2022). Association of Oxidative Stress-Induced Nucleic Acid Damage with Psychiatric Disorders in Adults: A Systematic Review and Meta-analysis. JAMA Psychiatry.

[11] Koweszko T., Gierus J., Zalewska A., Maciejczyk M., Waszkiewicz N., Szulc A. (2020). The Relationship between Suicide and Oxidative Stress in a Group of Psychiatric Inpatients. J. Clin. Med..

[12] Galli F., Bartolini D., Ronco C. (2024). Oxidative stress, defective proteostasis and immunometabolic complications in critically ill patients. Eur. J. Clin. Invest..

[13] Arabi Y., Jawdat D., Bouchama A., Tamim H., Tamimi W., Al-Balwi M., Al-Dorzi H.M., Sadat M., Afesh L., Lehe C. (2019). Oxidative stress, caloric intake and outcomes of critically ill patients. Clin. Nutr. ESPEN.

[14] Dresen E., Pimiento J.M., Patel J.J., Heyland D.K., Rice T.W., Stoppe C. (2023). Overview of oxidative stress and the role of micronutrients in critical illness. JPEN J. Parenter Enteral. Nutr..

[15] Kościuczuk U., Jakubów P., Czyżewska J., Knapp P., Rynkiewicz-Szczepańska E. (2022). Plasma brain-derived neurotrophic factor and opioid therapy: Results of pilot cross-sectional study. Clin. Med. Res..

[16] Mihaljevic O., Zivancevic-Simonovic S., Jovanovic D., Drakulic S.M., Vukajlovic J.T., Markovic A., Pirkovic M.S., Srejovic I., Jakovljevic V., Milosevic-Djordjevic O. (2023). Oxidative stress and DNA damage in critically ill patients with sepsis. Mutat Res. Genet. Toxicol Env. Mutagen.

[17] Fesharaki-Zadeh A. (2022). Oxidative Stress in Traumatic Brain Injury. Int. J. Mol. Sci..

[18] Rynkiewicz-Szczepanska E., Kosciuczuk U., Maciejczyk M. (2024). Total Antioxidant Status in Critically Ill Patients with Traumatic Brain Injury and Secondary Organ Failure-A Systematic Review. Diagnostics.

[19] Pryzmont M., Kosciuczuk U., Maciejczyk M. (2025). Biomarkers of traumatic brain injury: Narrative review and future prospects in neurointensive care. Front. Med..

[20] Khatri N., Thakur M., Pareek V., Kumar S., Sharma S., Datusalia A.K. (2018). Oxidative Stress: Major Threat in Traumatic Brain Injury. CNS Neurol. Disord. Drug. Targets.

[21] Rodríguez-Rodríguez A., Egea-Guerrero J.J., Murillo-Cabezas F., Carrillo-Vico A. (2014). Oxidative stress in traumatic brain injury. Curr. Med. Chem..

[22] Sies H. (2015). Oxidative stress: A concept in redox biology and medicine. Redox Biol..

[23] Forszt D., Gerreth K., Karpienko K., Zalewska A., Hojan K., Marchewka R., Bielas M., Maciejczyk M. (2025). Salivary chemokines and growth factors in patients with ischemic stroke. Sci. Rep..

[24] Erel O. (2005). A New automated colorimetric metod for measuring Total oxidant status. Clin. Biochem..

[25] Ghiselli A., Serafini M., Natella F., Scaccini C. (2000). Total antioxidant capacity as a tool to assess redox status: Critical view and experimental data. Free. Radic. Biol. Med..

[26] Silvestrini A., Meuci E., Ricerca B., Mancini A. (2023). Total antioxidant capacity: Biochemical aspects and clinical. Int. J. Mol. Sci..

[27] Munteanu I., Apetrei C. (2021). Analytical Methods used In determining antioxidant activity: A review. Int. J. Mole Sci..

[28] Grune T., Berger M. (2007). Markers of oxidative stress In ICU clinical settings: Present and future. Curr. Opin. Clin. Nutr. Metab. Care.

[29] Chuang C.C., Shiesh S.C., Chi C.H., Tu Y.F., Hor L.I., Shieh C.C., Chen M.F. (2006). Serum total antioxidant capacity reflects severity of illness in patients with severe sepsis. Crit. Care.

[30] Lorente L., Martín M.M., Pérez-Cejas A., Abreu-González P., López R., Ferreres J., Solé-Violán J., Labarta L., Díaz C., Palmero S. (2018). Serum total antioxidant capacity during the first week of sepsis and mortality. Obs. Study J. Crit. Care.

[31] Smykiewicz J., Tomasiuk R., Cemaga R., Buczkowski J., Maciejczyk M. (2025). Association of inflammation and protein carbamylation in patients with COVID-19. Front. Med..

[32] Ptaszyńska-Sarosiek I., Nesterowicz M., Gołaś E., Niemcunowicz-Janica A., Zalewska A., Żendzian-Piotrowska M., Maciejczyk M. (2025). Assessment of blood and urine total antioxidant and oxidative status in alcohol and acetone fatal poisonings. Sci. Rep..

[33] Neri M., Büttner A., Fineschi V. (2017). Brain Injury due to Mechanical Trauma and Ischemic-Hypoxic Insult: Biomarkers of Brain Injury and Oxidative Stress. Oxid. Med. Cell Longev..

[34] Abdul-Muneer P.M., Chandra N., Haorah J. (2015). Interactions of oxidative stress and neurovascular inflammation in the pathogenesis of traumatic brain injury. Mol. Neurobiol..

[35] Rincon F., Ghosh S., Dey S., Maltenfort M., Vibbert M., Urtecho J., McBride W., Moussouttas M., Bell R., Ratliff J.K. (2012). Impact of acute lung injury and acute respiratory distress syndrome after traumatic brain injury in the United States. Neurosurgery.

[36] Muballe K., Sewani-Rusike C., Longo-Mbenza B., Iputo J. (2019). Predictors of recovery in moderate to severe traumatic brain injury. J. Neurosurg..

[37] Lorente L., Martin M., Perez-Cejas A., Abreu-Gonzalez P., Ramos L., Argueso M., Caceres J., Sole-Violan J., Jimenez A. (2016). Association between Total antioxidant capacity and mortality in ischemic stroke patients. Ann. Intensive Care.

[38] Cojocaru I.M., Cojocaru M., Sapira V., Ionescu A. (2013). Evaluation of oxidative stress in patients with acute ischemic stroke. Rom. J. Intern. Med..

[39] Lorente L., Martin M., Almeida T., Gonzales P., Ramos L., Argueso M., Riano-Ruiz M., Sole-Violan J., Jimenez A. (2015). Total antioxidant capacity is associated with mortality of patients with severe traumatic brain injury. BMC Neurol..

[40] Lorente L., Martín M.M., Pérez-Cejas A., González-Rivero A.F., Abreu-González P., Ramos L., Argueso M., Solé-Violán J., Cáceres J.J., Jiménez A. (2020). Traumatic Brain Injury Patients Mortality and Serum Total Antioxidant Capacity. Brain Sci..

[41] Lorente L., Martín M.M., González-Rivero A.F., Pérez-Cejas A., Abreu-González P., Sabatel R., Ramos-Gómez L., Argueso M., Solé-Violán J., Cáceres J.J. (2020). Mortality prediction of ischemic stroke patients without thrombectomy by blood total antioxidant capacity. J. Integr. Neurosci..

[42] Lorente L., Martín M.M., Pérez-Cejas A., González-Rivero A.F., Sabatel R., Ramos-Gómez L., Argueso M., Solé-Violán J., Cáceres J.J., Jiménez A. (2021). High serum levels of TAC and early mortality in patients with spontaneous intracerebral haemorrhage. Neurol. Sci..

[43] Krenzlin H., Wesp D., Schmitt J., Frenz C., Kurz E., Masomi-Bornwasser J., Lotz J., Ringel F., Kerz T., Keric N. (2021). Decreased Superoxide Dismutase Concentrations (SOD) in Plasma and CSF and Increased Circulating Total Antioxidant Capacity (TAC) Are Associated with Unfavorable Neurological Outcome after Aneurysmal Subarachnoid Hemorrhage. J. Clin. Med..

[44] Servia L., Serrano J., Pampiona R., Badia M., Montserrat N., Portero-Otin M., Trujliano J. (2018). Location-dependent effects of trauma on oxidative stress in humans. PLoS ONE.

[45] Eroglu A. (2014). The effect of intravenous anesthetics on ischemia-reperfusion injury. BioMed Res. Int..

[46] Raczyńska A., Leszczyńska T., Skotnicki P., Koronowicz A. (2025). The Impact of Immunomodulatory Components Used in Clinical Nutrition—A Narrative Review. Nutrients.

[47] Alwarawrah Y., Kiernan K., MacIver N.J. (2018). Changes in Nutritional Status Impact Immune Cell Metabolism and Function. Front. Immunol..

[48] Peluso I., Raguzzini A. (2016). Salivary and urinary Total Antioxidant Capacity as biomarkers of oxidative stress in humans. Pathol. Res. Int..

[49] Paunikar S., Chakole V. (2024). Hyperoxia in Sepsis and Septic Shock: A Comprehensive Review of Clinical Evidence and Therapeutic Implications. Cureus.

[50] Eastman C.L., D’Ambrosio R., Ganesh T. (2020). Modulating neuroinflammation and oxidative stress to prevent epilepsy and improve outcomes after traumatic brain injury. Neuropharmacology.

[51] Quillinan N., Herson P.S., Traystman R.J. (2016). Neuropathophysiology of Brain Injury. Anesthesiol. Clin..

[52] Miliaraki M., Briassoulis P., Ilia S., Michalakakou K., Karakonstantakis T., Polonifi A., Bastaki K., Briassouli E., Vardas K., Pistiki A. (2022). Oxidant/Antioxidant Status Is Impaired in Sepsis and Is Related to Anti-Apoptotic, Inflammatory, and Innate Immunity Alterations. Antioxidants.

[53] Holland M.C., Mackersie R.C., Morabito D., Campbell A.R., Kivett V.A., Patel R., Erickson V.R., Pittet J.-F. (2003). The development of acute lung injury is associated with wose neurologic outcome in patients with severe traumatic brain injury. J. Trauma.

[54] Robba C., Zanier E.R., Soto C.L., Park S., Sonneville R., Helbolk R., Sarwal A., Newcombe V.F.J., van der Jagt M., Gunst J. (2024). Mastering the brain in critical conditions: An update. Intensive Care Med. Exp..

[55] Godoy D.A., Murillo-Cabezas F., Suarez J.I., Badenes R., Pelosi P., Robba C. (2023). “THE MANTLE” bundle for minimizing cerebral hypoxia in severe traumatic brain injury. Crit. Care.

[56] Maciejczyk M., Ptaszyńska-Sarosiek I., Niemcunowicz-Janica A., Szeremeta M., Waszkiewicz N., Kułak-Bejda A., Cwalina U., Nesterowicz M., Zalewska A. (2022). Do Circulating Redox Biomarkers Have Diagnostic Significance in Alcohol-Intoxicated People?. Int. J. Mol. Sci..

[57] Goodyear-Bruch C., Pierce J.D. (2002). Oxidative stress in critically ill patients. Am. J. Crit. Care.

[58] Sahoo D.K., Wong D., Patani A., Paital B., Yadav V.K., Patel A., Jergens A.E. (2024). Exploring the role of antioxidants in sepsis-associated oxidative stress: A comprehensive review. Front. Cell Infect. Microbiol..

[59] Bertozzi G., Ferrara M., Di Fazio A., Maiese A., Delogu G., Di Fazio N., Tortorella V., La Russa R., Fineschi V. (2024). Oxidative Stress in Sepsis: A Focus on Cardiac Pathology. Int. J. Mol. Sci..

[60] Bertozzi G., Ferrara M., Calvano M., Pascale N., Di Fazio A. (2024). Oxidative/Nitrosative Stress and Brain Involvement in Sepsis: A Relationship Supported by Immunohistochemistry. Medicina.

[61] Gao G., Wu X., Feng J., Hui J., Mao Q., Lecky F., Lingsma H., Maas A.I.R., Jiang J. (2020). Clinical characteristics and outcomes in patients with traumatic brain injury in China: A prospective, multicentre, longitudinal, observational study. Lancet.

[62] Mohideen K., Chandrasekaran K., Veeraraghavan H., Faizee S.H., Dhungel S., Ghosh S. (2023). Meta-Analysis of Assessment of Total Oxidative Stress and Total Antioxidant Capacity in Patients with Periodontitis. Dis. Markers.

